# Crystal structure of the thalidomide analog (3aR*,7aS*)-2-(2,6-dioxopiperidin-3-yl)hexa­hydro-1*H*-iso­indole-1,3(2*H*)-dione

**DOI:** 10.1107/S2056989018014317

**Published:** 2018-10-16

**Authors:** Yousef Hijji, Ellis Benjamin, Jerry P. Jasinski, Ray J. Butcher

**Affiliations:** a Department of Chemistry and Earth Sciences, Qatar University, Doha, Qatar; b Department of Chemistry, Richard Stockton College of New Jersey, Galloway, NJ 08205, USA; c Department of Chemistry, Keene State College, 229 Main Street, Keene NH 03435, USA; d Department of Chemistry, Howard University, 525 College Street NW, Washington, DC 20059, USA

**Keywords:** crystal structure, thalidomide analogs, pseudomerohedral twinning

## Abstract

The title compound, C_13_H_16_N_2_O_4_, crystallizes in the monoclinic centrosymmetric space group, *P*2_1_/*c*, with four mol­ecules in the asymmetric unit, thus there is no crystallographically imposed symmetry and it is a racemic mixture. The structure consists of a six-membered unsaturated ring bound to a five-membered pyrrolidine-2,5-dione ring N-bound to a six-membered piperidine-2,6-dione ring and thus has the same basic skeleton as thalidomide, except for the six-membered unsaturated ring substituted for the aromatic ring. In the crystal, the mol­ecules are linked into inversion dimers by *R* ^2^_2_(8) hydrogen bonding involving the N—H group. In addition, there are bifurcated C—H⋯O inter­actions involving one of the O atoms on the pyrrolidine-2,5-dione with graph-set notation *R* ^1^_2_(5). These inter­actions along with C—H⋯O inter­actions involving one of the O atoms on the piperidine-2,6-dione ring link the mol­ecules into a complex three-dimensional array. There is pseudomerohedral twinning present which results from a 180° rotation about the [100] reciprocal lattice direction and with a twin law of 1 0 0 0  0 0 0  [BASF 0.044 (1)].

## Abstract 2

The title compound, C_13_H_16_N_2_O_4_, consists of a six-membered unsaturated ring bound to a five-membered pyrrolidine-2,5-dione ring and N-bound to a six-membered piperidine-2,6-dione ring and thus has the same basic skeleton as thalidomide, except for the six-membered unsaturated ring substituted for the aromatic ring.

## Chemical context   

Thalidomide (**1**) is one of the most notorious drugs in pharmaceutical history because of the humanitarian disaster in the 1950s (Burley & Lenz, 1962 ▸; Stephans, 1988 ▸; Bartlett *et al.*, 2004 ▸; Wu *et al.*, 2005 ▸; Melchert & List, 2007 ▸). Thalidomide possesses a single stereogenic carbon in the glutarimide ring, and it is conceivable that the unexpected teratogenic side effects are ascribed to the (*S*)-enanti­omer of **1** (Blaschke *et al.*, 1979 ▸). However, this has been a matter of debate because considerable chiral inversion should take place during the incubation of enanti­omerically pure **1** (Nishimura *et al.*, 1994 ▸; Knoche & Blaschke, 1994 ▸; Wnendt *et al.*, 1996 ▸). Despite the tragic disaster, the unique biological properties of **1** prompted its return to the market in the 21st century for the treatment of multiple myeloma and leprosy (Matthews & McCoy, 2003 ▸; Hashimoto *et al.*, 2004 ▸; Franks *et al.*, 2004 ▸; Brennen *et al.*, 2004 ▸; Luzzio *et al.*, 2004 ▸; Sleijfer *et al.*, 2004 ▸; Kumar *et al.*, 2004 ▸; Hashimoto, 2008 ▸; Knobloch & Rüther, 2008 ▸). Furthermore, a large number of papers on novel medical uses of **1** continue to appear in the biological and medicinal literature (Matthews & McCoy, 2003 ▸; Hashimoto *et al.*, 2004 ▸; Franks *et al.*, 2004 ▸; Brennen *et al.*, 2004 ▸; Luzzio *et al.*, 2004 ▸; Sleijfer *et al.*, 2004 ▸; Kumar *et al.*, 2004 ▸; Hashimoto, 2008 ▸; Knobloch & Rüther, 2008 ▸).
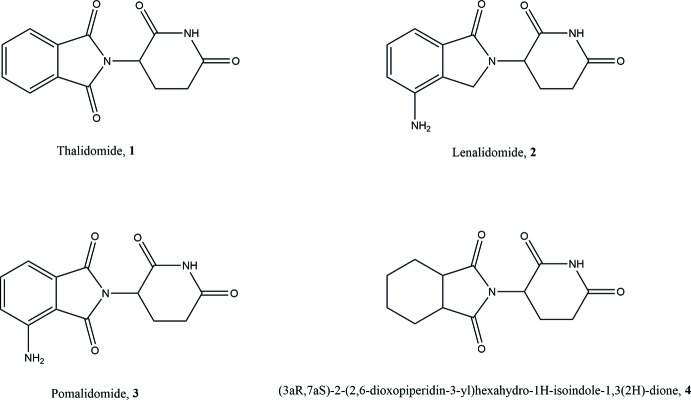


Thus, over the years, there has been increasing inter­est in thalidomide and its derivatives for the treatment of various hematologic malignancies (Singhal *et al.*, 1999 ▸; Raje & Anderson, 1999 ▸), solid tumors (Kumar *et al.*, 2002 ▸), and a variety of inflammatory and autoimmune diseases (Tseng *et al.*, 1996 ▸). Recent studies have uncovered a variety of mechanisms of thalidomide action. It was reported in 1991 that thalidomide is a selective inhibitor of tumor necrosis factor-α (TNF-α) production in lipopolysaccharide (LPS) stimulated human monocytes (Moreira *et al.*, 1993 ▸; Sampaio *et al.*, 1991 ▸). TNF-a is a key pro-inflammatory cytokine, and elevated levels have been linked with the pathology of a number of inflammatory and autoimmune diseases including rheumatoid arthritis, Crohn’s disease, aphthous ulcers, cachexia, graft *versus* host disease, asthma, ARDS and AIDS (Eigler *et al.*, 1997 ▸). Taken together, the immunomodulatory properties of thalidomide, which are dependent on the type of immune cell activated as well as the type of stimulus that the cell receives, provide a rationale for the mechanism of thalidomide action in the context of autoimmune and inflammatory disease states. Other pharmacologic activities of thalidomide include its inhibition of angiogenesis (D’Amato *et al.*, 1994 ▸) and its anti-cancer properties (Bartlett *et al.*, 2004 ▸). In the late 1990′s it was reported that thalidomide is efficacious for the treatment of multiple myeloma (MM), a hematological cancer caused by growth of tumor cells derived from the plasma cells in the bone marrow (Singhal *et al.*, 1999 ▸; Raje & Anderson, 1999 ▸).

A medicinal chemistry program to optimize the immunomodulatory properties of thalidomide and reduce its side-effects led to the discovery of lenalidomide (**2**), which is a potent immunomodulator that is ∼800 times more potent as an inhibitor of TNF-α in LPS-stimulated hPBMC (Muller *et al.*, 1999 ▸; Zeldis *et al.*, 2011 ▸). In the US, lenalidomide was approved by the FDA in 2005 for low- or inter­mediate-1-risk myelodysplastic

Structural optimization of thalidomide, **1** also led to the discovery of pomalidomide (**3**), which is tenfold more potent than lenalidomide as a TNF-a inhibitor and IL-2 stimulator (Muller *et al.*, 1999 ▸; Zeldis *et al.*, 2011 ▸). Pomalidomide is currently undergoing late-stage clinical development for the treatment of multiple myeloma and myeloproliferative neoplasm-associated myelofibrosis (Galustian & Dalgleish, 2011 ▸; Begna *et al.*, 2012 ▸). In clinical trials for multiple myeloma, pomalidomide has been shown to be effective in overcoming resistance to lenalidomide and thalidomide, as well as the proteosome inhibitor bortezomib (Schey & Ramasamy, 2011 ▸).

These studies have shown the efficacy of a continued search for more pharmacologically active analogs of thalidomide and its derivatives. Focus has previously been on modifying the basic thalidomide skeleton by changing its substituents. However, there have been very few studies on related derivatives where the six-membered ring is changed from an aromatic to an unsaturated ring. In view of the wide inter­est in these types of compounds for their pharmacological activities, the structure of (3a*R*,7a*S*)-2-(2,6-dioxopiperidin-3-yl)hexa­hydro-1*H*-iso­indole-1,3(2*H*)-dione, **4**, is reported where the only change to thalidomide is the substitution of an unsaturated six-membered for the aromatic ring.

As a result of this inter­est in thalidomide, the crystal structure of this mol­ecule in both the racemic and enanti­o­merically pure forms have been determined multiple times (Lovell, 1970 ▸, 1971 ▸; Reepmeyer *et al.*, 1994 ▸; Allen & Trotter, 1971 ▸; Caira *et al.*, 1994 ▸; Suzuki *et al.*, 2010 ▸; Maeno *et al.*, 2015 ▸). Two polymorphs of the racemic derivative have been determined crystallizing in the space groups *P*2_1_/*n* (Allen & Trotter, 1971 ▸; Suzuki *et al.*, 2010 ▸; Maeno *et al.*, 2015 ▸) and *P*2_1_/*c* (Lovell, 1970 ▸) or *C*2/*c* (Reepmeyer *et al.*, 1994 ▸; Caira *et al.*, 1994 ▸). The crystal packing in the *C*2/*c* structure is determined by inter­molecular N–H⋯O hydrogen bonding that is more extensive than that reported for the racemate of thalidomide crystallizing in space group *P*2_1_/*n*.

## Structural commentary   

**Figure 1:**
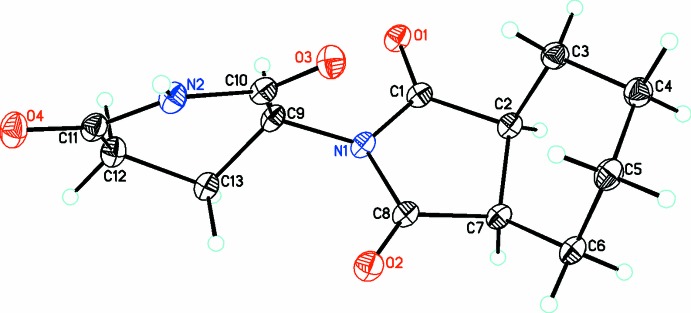
The molecular structure of the title compound **4**, with the atom-numbering scheme. Atomic displacement parameters are drawn at the 30% probability level.

The title compound, C_13_H_16_N_2_O_4_, **4** (Fig. 1 ▸), crystallizes in the monoclinic centrosymmetric space group, *P*2_1_/*c*, with four mol­ecules in the asymmetric unit, thus there is no crystallographically imposed symmetry and it is a racemic mixture. The structure consists of a six-membered unsaturated ring bound to a five-membered pyrrolidine-2,5-dione ring N-bound to a six-membered piperidine-2,6-dione ring and thus has the same basic skeleton as thalidomide, **1**, except for the six-membered unsaturated ring substituted for the aromatic ring. In the five-membered pyrrolidine-2,5-dione ring, the atoms O1, C1, N1, C8 and O2 form a plane (r.m.s. deviation of fitted atoms = 0.0348 Å) with C2 and C7 deviating from this plane by −0.186 (7) and 0.219 (7) Å, respectively. The ring itself adopts a conformation in which it is twisted about the C2–C7 axis [*P* = 257.4 (5) and τ = 22.5 (2); Rao *et al.*, 1981 ▸]. In the six-membered piperidine-2,6-dione ring, the group, O3, C10, N2, C11and O4 is also planar (r.m.s. deviation of fitted atoms = 0.0042 Å). The cyclo­hexane ring adopts a chair conformation [puckering parameters *Q* = 0.536 (3), θ = 157.7 (3)° and φ = 324.2 (8)°; Boeyens, 1978 ▸). Otherwise, the metrical parameters for all bonds are in the standard range for such structures.

## Supra­molecular features   

**Figure 2:**
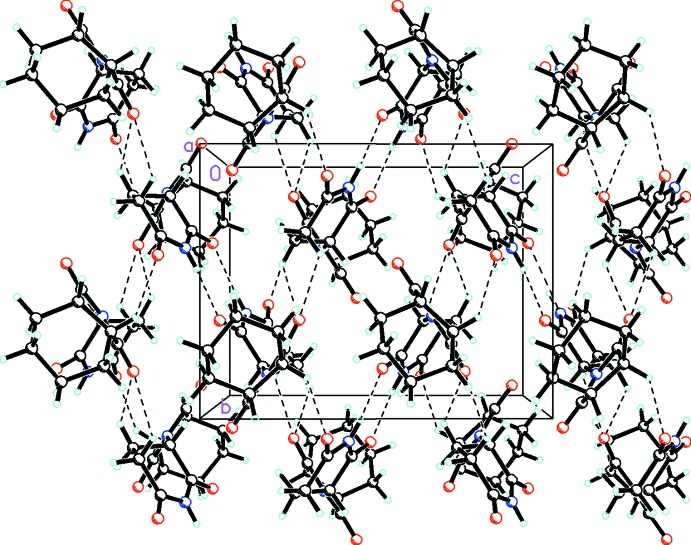
Packing diagram viewed along the *a* axis showing the extensive N—H⋯O and C—H⋯O inter­actions (drawn as dashed lines) linking the mol­ecules into a complex three-dimensional array.

Similarly to the hydrogen-bonding patterns found in both the enanti­omerically pure form of thalidomide (Lovell, 1971 ▸; Maeno *et al.*, 2015 ▸) and the racemic *P*2_1_/*n* polymorph (Allen & Trotter, 1971 ▸; Suzuki *et al.*, 2010 ▸; Maeno *et al.*, 2015 ▸), the mol­ecules of the title compound are linked into inversion dimers by 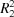
(8) (Etter *et al.*, 1990 ▸) hydrogen bonding (Table 1 ▸) involving the N—H group as shown in Fig. 2 ▸. In addition, there are bifurcated C—H⋯O inter­actions involv­ing O2 with graph-set notation 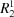
(5). These inter­actions, along with C—H⋯O inter­actions involving O4, link the mol­ecules into a complex three-dimensional array.

## Database survey   

A search of the Cambridge Structural Database (CSD version 5.39; Groom *et al.*, 2016 ▸) using a skeleton containing the three rings as in thalidomide but without the ketone substituents gave 39 hits but not a single example where the six-membered aromatic ring in the isoindoline moiety is changed to an unsaturated six-membered ring.

## Synthesis and crystallization   

Some details of the synthesis have been previously reported (Benjamin & Hijji, 2017 ▸). *cis*-1,2-Cyclo­hexane di­carb­oxy­lic acid anhydride (0.10 g, 0.65 mmol), glutamic acid (0.095 g, 0.65 mmol), DMAP (0.02 g, 0.16 mmol), and ammonium chloride (NH_4_Cl) (0.040 g, 0.75 mmol) were mixed thoroughly in a CEM-sealed vial with a magnetic stirrer. The sample was heated for 6 min at 423 K in a CEM Discover microwave powered at 150 W. It was then cooled rapidly to 313 K and dissolved in 15 ml of (1:1) ethyl acetate:acetone. The organic layer was washed with 2× 10 ml of distilled water and dried over sodium sulfate (anhydrous). The organic layer was concentrated under vacuum and precipitated with hexa­nes (30 ml) affording a white solid. Crystals suitable for X-ray experiments were grown by slow evaporation of an ethyl acetate/acetone (1:1) solution. M.p. 463–465 K, (0.12 g, 70%). ^1^H NMR (400 MHz, DMSO-*d_6_*) δ 11.0 (*s*, 1 H, NH), 4.9 (*dd*, 1 H, 12.5, 5.5 Hz, CHCO), 3.0 (*m*, 1 H), 2.8 (*m*, 1 H), 2.8 (*m*, 1 H), 2.5 (*m*, 1 H), 1.9 (*m*, 1 H), 1.7 (*m*, 3 H),, 1.6 (*m*, 1 H), 1.4 (*m*, 4 H); ^13^C NMR (100 MHz, DMSO-*d_6_*) 178.8 (C=O), 178.7 (C=O), 172.7 (C=O), 169.4 (C=O), 48.7 (CH), 39.1 (CH), 38.8 (CH), 30.7 (CH_2_), 23.1 (CH_2_), 22.9 (CH_2_), 21.1 (CH_2_), 21.05 (CH_2_), 21.00 (CH_2_); MS 264 (*M* ^+^); 236, 210, 179, 154, 112, 82, 67, 54, 41.

## Refinement   

Crystal data, data collection and structure refinement details are summarized in Table 2 ▸. H atoms were positioned geometrically and treated as riding on their parent atoms and refined with C—H distances of 0.99–1.00 Å and *U*_iso_(H) = 1.2*U*_eq_(C). The H attached to N2 was refined isotropically. There is pseudomerohedral twinning present, which results from a 180° rotation about the [100] reciprocal lattice direction and with a twin law of 1 0 0 0 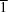 0 0 0 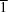 [BASF 0.044 (1)].

## Supplementary Material

Crystal structure: contains datablock(s) I. DOI: 10.1107/S2056989018014317/lh5881sup1.cif

Structure factors: contains datablock(s) I. DOI: 10.1107/S2056989018014317/lh5881Isup2.hkl

Supporting information file. DOI: 10.1107/S2056989018014317/lh5881Isup3.cml

CCDC reference: 1872551

Additional supporting information:  crystallographic information; 3D view; checkCIF report

## Figures and Tables

**Table 1:** Hydrogen-bond geometry (Å, °)

*D*—H⋯*A*	*D*—H	H⋯*A*	*D*⋯*A*	*D*—H⋯*A*
N2—H2*N*⋯O3^i^	0.88 (5)	2.07 (5)	2.928 (3)	165 (4)
C7—H7*A*⋯O4^ii^	1.00	2.42	3.150 (3)	129
C9—H9*A*⋯O1^iii^	1.00	2.65	3.385 (3)	130
C12—H12*A*⋯O2^ii^	0.99	2.53	3.143 (3)	120
C13—H13*A*⋯O2	0.99	2.56	3.142 (3)	118
C13—H13*B*⋯O2^ii^	0.99	2.52	3.163 (3)	122

Symmetry codes: (i) 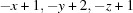; (ii) 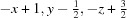; (iii) 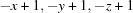.

**Table 2:** Experimental details

Crystal data	
Chemical formula	C_13_H_16_N_2_O_4_
*M*_r_	264.28
Crystal system, space group	Monoclinic, *P*2_1_/*c*
Temperature (K)	123
*a*, *b*, *c* (Å)	11.4519 (3), 9.2370 (3), 11.8727 (4)
β (°)	90.475 (3)
*V* (Å^3^)	1255.87 (7)
*Z*	4
Radiation type	Cu *K*α
μ (mm^−1^)	0.87
Crystal size (mm)	0.42 × 0.34 × 0.18

Data collection	
Diffractometer	Rigaku Oxford Diffraction Xcalibur, Ruby, Gemini
Absorption correction	Multi-scan (*CrysAlis PRO*; Rigaku OD, 2012 ▸)
*T*_min_, *T*_max_	0.822, 1.000
No. of measured, independent and observed [*I* > 2σ(*I*)] reflections	9733, 2626, 2572
*R*_int_	0.024
(sin θ/λ)_max_ (Å^−1^)	0.633

Refinement	
*R*[*F* ^2^ > 2σ(*F* ^2^)], *wR*(*F* ^2^), *S*	0.066, 0.208, 1.19
No. of reflections	2626
No. of parameters	177
H-atom treatment	H atoms treated by a mixture of independent and constrained refinement
Δρ_max_, Δρ_min_ (e Å^−3^)	0.33, −0.35

Computer programs: *CrysAlis PRO* (Rigaku OD, 2012 ▸), *SHELXS97* and *SHELXTL* (Sheldrick, 2008 ▸) and *SHELXL2018/3* (Sheldrick, 2015 ▸).

## supplementary crystallographic information

### Crystal data

**Table:**

C_13_H_16_N_2_O_4_	*F*(000) = 560
*M**_r_* = 264.28	*D*_x_ = 1.398 Mg m^−^^3^
Monoclinic, *P*2_1_/*c*	Cu *K*α radiation, λ = 1.54178 Å
*a* = 11.4519 (3) Å	Cell parameters from 7629 reflections
*b* = 9.2370 (3) Å	θ = 3.7–77.3°
*c* = 11.8727 (4) Å	µ = 0.87 mm^−^^1^
β = 90.475 (3)°	*T* = 123 K
*V* = 1255.87 (7) Å^3^	Prism, colorless
*Z* = 4	0.42 × 0.34 × 0.18 mm

### Data collection

**Table:**

Rigaku Oxford Diffraction Xcalibur, Ruby, Gemini diffractometer	2572 reflections with *I* > 2σ(*I*)
Detector resolution: 10.5081 pixels mm^-1^	*R*_int_ = 0.024
ω scans	θ_max_ = 77.5°, θ_min_ = 3.7°
Absorption correction: multi-scan (CrysAlisPro; Rigaku OD, 2012)	*h* = −9→14
*T*_min_ = 0.822, *T*_max_ = 1.000	*k* = −10→11
9733 measured reflections	*l* = −14→14
2626 independent reflections	

### Refinement

**Table:**

Refinement on *F*^2^	Primary atom site location: structure-invariant direct methods
Least-squares matrix: full	Secondary atom site location: difference Fourier map
*R*[*F*^2^ > 2σ(*F*^2^)] = 0.066	Hydrogen site location: mixed
*wR*(*F*^2^) = 0.208	H atoms treated by a mixture of independent and constrained refinement
*S* = 1.19	*w* = 1/[σ^2^(*F*_o_^2^) + (0.1179*P*)^2^ + 1.1244*P*] where *P* = (*F*_o_^2^ + 2*F*_c_^2^)/3
2626 reflections	(Δ/σ)_max_ < 0.001
177 parameters	Δρ_max_ = 0.33 e Å^−^^3^
0 restraints	Δρ_min_ = −0.35 e Å^−^^3^

### Special details

**Table:**

Geometry. All esds (except the esd in the dihedral angle between two l.s. planes) are estimated using the full covariance matrix. The cell esds are taken into account individually in the estimation of esds in distances, angles and torsion angles; correlations between esds in cell parameters are only used when they are defined by crystal symmetry. An approximate (isotropic) treatment of cell esds is used for estimating esds involving l.s. planes.
Refinement. Refined as a two-component twin

### Fractional atomic coordinates and isotropic or equivalent isotropic displacement parameters (Å^2^)

**Table:**

	*x*	*y*	*z*	*U*_iso_*/*U*_eq_	
O1	0.66960 (17)	0.4309 (2)	0.56402 (17)	0.0291 (4)	
O2	0.67111 (18)	0.8499 (2)	0.76606 (18)	0.0305 (5)	
O3	0.58392 (17)	0.8448 (2)	0.51862 (17)	0.0299 (5)	
O4	0.21720 (17)	0.9000 (2)	0.64578 (19)	0.0339 (5)	
N1	0.64254 (19)	0.6373 (2)	0.66901 (18)	0.0228 (5)	
N2	0.4008 (2)	0.8685 (2)	0.58537 (19)	0.0263 (5)	
H2N	0.393 (4)	0.952 (5)	0.550 (3)	0.043 (10)*	
C1	0.7100 (2)	0.5317 (3)	0.6160 (2)	0.0233 (5)	
C2	0.8368 (2)	0.5762 (3)	0.6283 (2)	0.0240 (5)	
H2A	0.886927	0.491448	0.648892	0.029*	
C3	0.8713 (2)	0.6388 (3)	0.5124 (2)	0.0288 (6)	
H3A	0.893112	0.558146	0.461791	0.035*	
H3B	0.802837	0.688401	0.478581	0.035*	
C4	0.9729 (2)	0.7454 (3)	0.5201 (2)	0.0311 (6)	
H4A	1.043151	0.695475	0.549779	0.037*	
H4B	0.990952	0.783222	0.444218	0.037*	
C5	0.9407 (2)	0.8704 (3)	0.5979 (2)	0.0295 (6)	
H5A	1.003226	0.944367	0.597275	0.035*	
H5B	0.867389	0.916344	0.571200	0.035*	
C6	0.9248 (2)	0.8128 (3)	0.7171 (2)	0.0278 (6)	
H6A	0.899178	0.893048	0.766341	0.033*	
H6B	1.001037	0.777727	0.746054	0.033*	
C7	0.8356 (2)	0.6895 (3)	0.7240 (2)	0.0236 (5)	
H7A	0.850009	0.636936	0.796398	0.028*	
C8	0.7103 (2)	0.7412 (3)	0.7241 (2)	0.0235 (5)	
C9	0.5186 (2)	0.6584 (3)	0.6460 (2)	0.0236 (5)	
H9A	0.491323	0.576348	0.597625	0.028*	
C10	0.5061 (2)	0.7980 (3)	0.5776 (2)	0.0239 (5)	
C11	0.3047 (2)	0.8261 (3)	0.6481 (2)	0.0264 (5)	
C12	0.3171 (2)	0.6889 (3)	0.7153 (2)	0.0285 (6)	
H12A	0.288711	0.606388	0.669460	0.034*	
H12B	0.267804	0.695498	0.783153	0.034*	
C13	0.4435 (2)	0.6608 (3)	0.7512 (2)	0.0260 (5)	
H13A	0.470638	0.737979	0.802975	0.031*	
H13B	0.449314	0.566826	0.790999	0.031*	

### Atomic displacement parameters (Å^2^)

**Table:**

	*U*^11^	*U*^22^	*U*^33^	*U*^12^	*U*^13^	*U*^23^
O1	0.0298 (9)	0.0199 (9)	0.0374 (10)	−0.0011 (7)	−0.0051 (8)	−0.0043 (7)
O2	0.0306 (10)	0.0200 (9)	0.0407 (11)	0.0001 (7)	−0.0028 (8)	−0.0059 (7)
O3	0.0280 (10)	0.0269 (10)	0.0347 (10)	0.0042 (7)	0.0006 (8)	0.0055 (7)
O4	0.0266 (9)	0.0273 (10)	0.0478 (12)	0.0052 (8)	−0.0031 (8)	−0.0019 (9)
N1	0.0226 (10)	0.0164 (9)	0.0294 (10)	0.0004 (8)	−0.0062 (8)	0.0003 (8)
N2	0.0260 (11)	0.0189 (10)	0.0337 (11)	0.0043 (8)	−0.0044 (9)	0.0023 (9)
C1	0.0265 (12)	0.0173 (11)	0.0259 (11)	0.0017 (9)	−0.0049 (9)	0.0024 (9)
C2	0.0245 (11)	0.0177 (11)	0.0297 (12)	0.0005 (9)	−0.0039 (9)	0.0002 (9)
C3	0.0297 (13)	0.0276 (13)	0.0291 (13)	−0.0027 (10)	−0.0008 (10)	−0.0019 (10)
C4	0.0306 (13)	0.0319 (14)	0.0309 (13)	−0.0046 (11)	−0.0008 (10)	0.0011 (10)
C5	0.0286 (13)	0.0252 (13)	0.0345 (14)	−0.0050 (10)	−0.0035 (10)	0.0027 (10)
C6	0.0250 (12)	0.0275 (12)	0.0309 (13)	−0.0053 (10)	−0.0045 (10)	−0.0006 (10)
C7	0.0240 (11)	0.0216 (11)	0.0253 (11)	−0.0020 (9)	−0.0039 (9)	0.0014 (9)
C8	0.0250 (11)	0.0195 (11)	0.0258 (11)	−0.0010 (9)	−0.0043 (9)	0.0017 (9)
C9	0.0219 (11)	0.0170 (11)	0.0319 (12)	0.0015 (8)	−0.0070 (9)	−0.0005 (9)
C10	0.0253 (11)	0.0180 (11)	0.0283 (11)	0.0021 (9)	−0.0054 (9)	−0.0006 (9)
C11	0.0242 (12)	0.0213 (12)	0.0336 (13)	0.0007 (9)	−0.0054 (10)	−0.0053 (10)
C12	0.0245 (12)	0.0217 (12)	0.0393 (14)	−0.0016 (9)	−0.0017 (10)	0.0001 (10)
C13	0.0239 (12)	0.0218 (12)	0.0322 (13)	−0.0007 (9)	−0.0034 (10)	0.0030 (9)

### Geometric parameters (Å, º)

**Table:**

O1—C1	1.207 (3)	C4—H4B	0.9900
O2—C8	1.208 (3)	C5—C6	1.525 (4)
O3—C10	1.217 (3)	C5—H5A	0.9900
O4—C11	1.213 (3)	C5—H5B	0.9900
N1—C8	1.394 (3)	C6—C7	1.532 (3)
N1—C1	1.397 (3)	C6—H6A	0.9900
N1—C9	1.456 (3)	C6—H6B	0.9900
N2—C10	1.374 (3)	C7—C8	1.513 (3)
N2—C11	1.390 (4)	C7—H7A	1.0000
N2—H2N	0.88 (5)	C9—C13	1.522 (4)
C1—C2	1.515 (3)	C9—C10	1.531 (3)
C2—C7	1.544 (3)	C9—H9A	1.0000
C2—C3	1.548 (4)	C11—C12	1.503 (4)
C2—H2A	1.0000	C12—C13	1.528 (4)
C3—C4	1.527 (4)	C12—H12A	0.9900
C3—H3A	0.9900	C12—H12B	0.9900
C3—H3B	0.9900	C13—H13A	0.9900
C4—C5	1.525 (4)	C13—H13B	0.9900
C4—H4A	0.9900		
			
C8—N1—C1	112.6 (2)	C5—C6—H6B	108.9
C8—N1—C9	122.3 (2)	C7—C6—H6B	108.9
C1—N1—C9	123.4 (2)	H6A—C6—H6B	107.8
C10—N2—C11	127.0 (2)	C8—C7—C6	113.4 (2)
C10—N2—H2N	118 (3)	C8—C7—C2	103.24 (19)
C11—N2—H2N	115 (3)	C6—C7—C2	117.1 (2)
O1—C1—N1	123.9 (2)	C8—C7—H7A	107.5
O1—C1—C2	128.4 (2)	C6—C7—H7A	107.5
N1—C1—C2	107.5 (2)	C2—C7—H7A	107.5
C1—C2—C7	103.9 (2)	O2—C8—N1	123.9 (2)
C1—C2—C3	105.49 (19)	O2—C8—C7	128.2 (2)
C7—C2—C3	113.9 (2)	N1—C8—C7	107.8 (2)
C1—C2—H2A	111.1	N1—C9—C13	113.9 (2)
C7—C2—H2A	111.1	N1—C9—C10	107.4 (2)
C3—C2—H2A	111.1	C13—C9—C10	111.9 (2)
C4—C3—C2	112.8 (2)	N1—C9—H9A	107.8
C4—C3—H3A	109.0	C13—C9—H9A	107.8
C2—C3—H3A	109.0	C10—C9—H9A	107.8
C4—C3—H3B	109.0	O3—C10—N2	121.2 (2)
C2—C3—H3B	109.0	O3—C10—C9	122.6 (2)
H3A—C3—H3B	107.8	N2—C10—C9	116.2 (2)
C5—C4—C3	109.7 (2)	O4—C11—N2	119.1 (2)
C5—C4—H4A	109.7	O4—C11—C12	124.1 (3)
C3—C4—H4A	109.7	N2—C11—C12	116.8 (2)
C5—C4—H4B	109.7	C11—C12—C13	112.1 (2)
C3—C4—H4B	109.7	C11—C12—H12A	109.2
H4A—C4—H4B	108.2	C13—C12—H12A	109.2
C6—C5—C4	109.2 (2)	C11—C12—H12B	109.2
C6—C5—H5A	109.8	C13—C12—H12B	109.2
C4—C5—H5A	109.8	H12A—C12—H12B	107.9
C6—C5—H5B	109.8	C9—C13—C12	108.3 (2)
C4—C5—H5B	109.8	C9—C13—H13A	110.0
H5A—C5—H5B	108.3	C12—C13—H13A	110.0
C5—C6—C7	113.2 (2)	C9—C13—H13B	110.0
C5—C6—H6A	108.9	C12—C13—H13B	110.0
C7—C6—H6A	108.9	H13A—C13—H13B	108.4
			
C8—N1—C1—O1	179.5 (2)	C9—N1—C8—C7	−174.9 (2)
C9—N1—C1—O1	−15.2 (4)	C6—C7—C8—O2	−34.8 (4)
C8—N1—C1—C2	−5.3 (3)	C2—C7—C8—O2	−162.5 (3)
C9—N1—C1—C2	160.0 (2)	C6—C7—C8—N1	147.1 (2)
O1—C1—C2—C7	−167.9 (2)	C2—C7—C8—N1	19.4 (3)
N1—C1—C2—C7	17.2 (2)	C8—N1—C9—C13	−67.6 (3)
O1—C1—C2—C3	72.0 (3)	C1—N1—C9—C13	128.4 (2)
N1—C1—C2—C3	−102.9 (2)	C8—N1—C9—C10	56.9 (3)
C1—C2—C3—C4	155.2 (2)	C1—N1—C9—C10	−107.1 (3)
C7—C2—C3—C4	41.9 (3)	C11—N2—C10—O3	179.1 (2)
C2—C3—C4—C5	−58.7 (3)	C11—N2—C10—C9	−0.4 (4)
C3—C4—C5—C6	64.9 (3)	N1—C9—C10—O3	25.9 (3)
C4—C5—C6—C7	−55.2 (3)	C13—C9—C10—O3	151.6 (2)
C5—C6—C7—C8	−80.1 (3)	N1—C9—C10—N2	−154.6 (2)
C5—C6—C7—C2	40.0 (3)	C13—C9—C10—N2	−28.9 (3)
C1—C2—C7—C8	−21.7 (2)	C10—N2—C11—O4	−179.6 (2)
C3—C2—C7—C8	92.6 (2)	C10—N2—C11—C12	0.4 (4)
C1—C2—C7—C6	−147.1 (2)	O4—C11—C12—C13	−151.1 (3)
C3—C2—C7—C6	−32.8 (3)	N2—C11—C12—C13	28.9 (3)
C1—N1—C8—O2	172.5 (2)	N1—C9—C13—C12	178.0 (2)
C9—N1—C8—O2	6.9 (4)	C10—C9—C13—C12	55.9 (3)
C1—N1—C8—C7	−9.4 (3)	C11—C12—C13—C9	−56.1 (3)

### Hydrogen-bond geometry (Å, º)

**Table:**

*D*—H···*A*	*D*—H	H···*A*	*D*···*A*	*D*—H···*A*
N2—H2*N*···O3^i^	0.88 (5)	2.07 (5)	2.928 (3)	165 (4)
C7—H7*A*···O4^ii^	1.00	2.42	3.150 (3)	129
C9—H9*A*···O1^iii^	1.00	2.65	3.385 (3)	130
C12—H12*A*···O2^ii^	0.99	2.53	3.143 (3)	120
C13—H13*A*···O2	0.99	2.56	3.142 (3)	118
C13—H13*B*···O2^ii^	0.99	2.52	3.163 (3)	122

Symmetry codes: (i) −*x*+1, −*y*+2, −*z*+1; (ii) −*x*+1, *y*−1/2, −*z*+3/2; (iii) −*x*+1, −*y*+1, −*z*+1.
